# The Case for Exploiting Cross-Species Epitopes in Malaria Vaccine Design

**DOI:** 10.3389/fimmu.2020.00335

**Published:** 2020-02-27

**Authors:** Catherine J. Mitran, Stephanie K. Yanow

**Affiliations:** ^1^School of Public Health, University of Alberta, Edmonton, AB, Canada; ^2^Department of Medical Microbiology and Immunology, University of Alberta, Edmonton, AB, Canada

**Keywords:** malaria, *Plasmodium*, heterologous, cross-species, immunity, vaccines, epitopes

## Abstract

The infection dynamics between different species of *Plasmodium* that infect the same human host can both suppress and exacerbate disease. This could arise from inter-parasite interactions, such as competition, from immune regulation, or both. The occurrence of protective, cross-species (heterologous) immunity is an unlikely event, especially considering that strain-transcending immunity within a species is only partial despite lifelong exposure to that species. Here we review the literature in humans and animal models to identify the contexts where heterologous immunity can arise, and which antigens may be involved. From the perspective of vaccine design, understanding the mechanisms by which exposure to an antigen from one species can elicit a protective response to another species offers an alternative strategy to conventional approaches that focus on immunodominant antigens within a single species. The underlying hypothesis is that certain epitopes are conserved across evolution, in sequence or in structure, and shared in antigens from different species. Vaccines that focus on conserved epitopes may overcome the challenges posed by polymorphic immunodominant antigens; but to uncover these epitopes requires approaches that consider the evolutionary history of protein families across species. The key question for vaccinologists will be whether vaccines that express these epitopes can elicit immune responses that are functional and contribute to protection against *Plasmodium* parasites.

## Introduction

**FIGURE 1:**
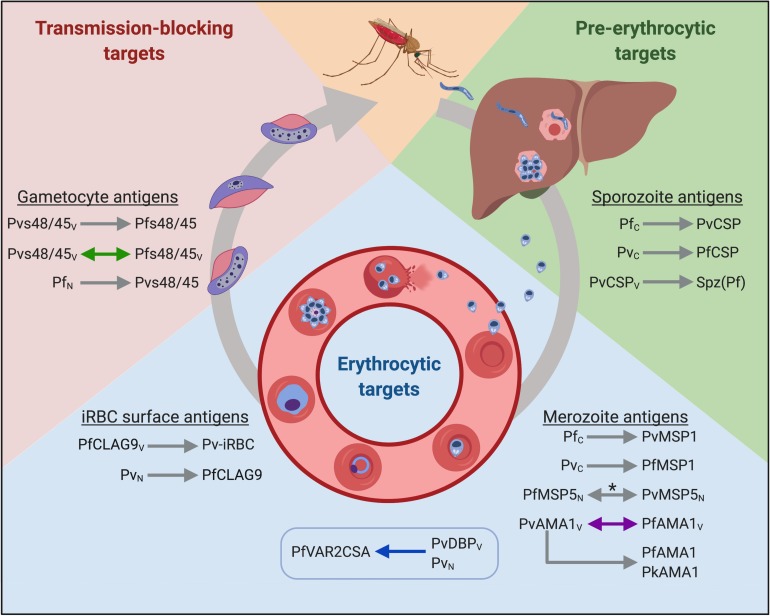
Putative cross-species vaccine candidates at different stages of the parasite life cycle. Arrowheads indicate the direction of cross-reactivity and double arrowheads show reciprocal cross-reactivity. Gray arrows denote immunological cross-reactivity, but unknown functional activity; purple arrows denote that heterologous function was not demonstrated; blue arrows denote that heterologous function was demonstrated, and green arrows denote cross-boosting following heterologous vaccination. The box indicates heterologous cross-stage reactivity (antibodies to the merozoite antigen recognize an iRBC surface antigen). Spz(Pf) = *P. falciparum* sporozoites. Subscript letters denote route of exposure to parasite or antigen; C = Controlled human malaria infections (CHMI); V = exposure through vaccination; N = natural infection. *Antigen recognition was blocked by heterologous antigen in a subset of samples from co-exposed individuals. Created with Biorender.com.

A malaria vaccine would have a tremendous impact on vulnerable populations, with the potential to save nearly half a million lives annually and prevent over 200 million cases (1). Yet the development of an efficacious vaccine remains elusive. One of the biggest challenges facing malaria vaccine development is the complex life cycle of the parasite (Figure 1). The sporozoite form of the parasite invades hepatocytes in the liver, undergoes schizogony, and then enters the blood stage. In the blood, some of the parasites differentiate to form gametocytes that can be taken up by mosquitoes during a blood meal, resulting in onward parasite transmission. The challenge to vaccinologists is that the parasite expresses antigens that are largely stage-specific during its lifecycle and no single defining vaccine target or even whole organism vaccine can protect against all stages. Despite this, there are multiple opportunities for vaccines to interrupt the parasite life cycle (2). A vaccine that prevents sporozoite colonization of hepatocytes could protect individuals from *Plasmodium* infection, while a vaccine targeting the blood stage could curb the clinical manifestation of disease, and a gametocyte-targeting vaccine could block transmission to mosquitoes.

Of the six species of *Plasmodium* that infect humans, *P. falciparum* causes the greatest mortality and morbidity worldwide (1). In high transmission settings, millions of young children are at risk of dying from severe falciparum malaria until they acquire immunity to severe disease later in childhood. As such, current vaccine efforts are largely focused on *P. falciparum*. Only one licensed vaccine exists, RTS,S and this vaccine is currently undergoing pilot roll-out in several African countries. The target of RTS,S is the surface circumsporozoite surface protein (CSP) that is expressed on the surface of *P. falciparum* sporozoites (3). While this vaccine aims to prevent liver stage infection, the results from earlier vaccine trials suggest good immunity in the first six months but then a significant waning of immunity over time, resulting in poor long-term vaccine efficacy (3). This is likely due in part to the low dose of sporozoites inoculated by mosquitoes that fails to reactivate memory B-cells, combined with antigenic polymorphisms in the T-cell epitopes of the CSP (4). Other vaccines target *P. falciparum* blood stage antigens and they also face significant challenges, primarily due to antigenic polymorphisms that reduce the efficacy of allele-specific vaccines against natural infections (5).

The limitations of current experimental vaccines may reflect a shortcoming in the traditional approach to antigen discovery (6). Candidates, particularly blood stage antigens, are often identified as targets of neutralizing antibodies in immune sera; but the corollary is that this strategy selects for immunodominant epitopes that are under strong immune selection, and consequently, are highly polymorphic. Incorporating conserved and cryptic epitopes (epitopes not normally exposed to the immune system) into vaccines may overcome these challenges.

Here we consider whether epitopes conserved across species can be exploited in vaccine design. This idea may seem heretical given the absence of sterile immunity following lifelong exposure to a single species, and our understanding that the immune response to malaria is largely considered strain-specific. In fact, cross-species immunity has doubtlessly been selected *against* due to the co-circulation of multiple *Plasmodium* species competing for the same human host. Competition between parasites likely resulted in the evolution of different virulence and life cycle strategies as a form of mutual adaptation, and within these species-specific adaptations arose antigenic diversity in virulence genes of that parasite. Nevertheless, the shared evolutionary history among the six species of *Plasmodium* purports that many proteins will be homologous in origin, with common structures and/or functions. As such, it is likely that there are subdominant or even immunologically cryptic epitopes that remain conserved across multiple species. As a vaccine strategy, this presents an opportunity to direct the immune response against these conserved epitopes and exploit them in a cross-species malaria vaccine.

In this review, we discuss the evidence for immunological cross-reactivity between *Plasmodium* species and the rationale for considering a cross-species vaccine approach. We define heterologous immunity and cross-reactivity as immunological interactions between two different *Plasmodium* species and not between two strains of the same species. We first consider the clinical outcomes of natural infection in areas co-endemic for multiple species, deliberate human infection studies, and infections in animal models. Next we describe the parasite-specific immune responses to different species of *Plasmodium* and the antigens that may mediate cross-species immunity. Lastly, we provide a rationale for mapping conserved epitopes in antigens from different species and developing these epitopes as vaccine candidates.

## Observations From Naturally Exposed Populations

Interactions between different species of *Plasmodium* are evident from a number of epidemiological studies of naturally exposed populations [reviewed in (7, 8)]. These are often reported as negative interactions, where co-infection with two species exacerbated disease (7), or provide no evidence of interaction at all - infection with one species had no demonstrable impact on the risk or severity of infection from another species (7). Yet concurrent studies from South Asia and Oceania gave rise to findings in support of cross-species immunity (9–11). In particular, there is compelling data that infection with *P. vivax* confers a degree of clinical protection against *P. falciparum*. This was observed in a prospective study in Sri Lanka, where the severity of symptoms from *P. falciparum* infection was lessened following a *P. vivax* infection, inferred as “clinical tolerance” to the more virulent species (9). Further support for this phenomenon was garnered from cross-sectional and longitudinal studies in Vanuatu where the incidence of severe malaria (severe anemia and cerebral malaria) was much lower than expected for an area hyperendemic for *P. falciparum* and *P. vivax* (10). The authors proposed that cross-species immunity may contribute to clinical protection and impact the infection dynamics of these two species (12). Subsequent data from a large-scale prospective analysis of health-center morbidity in Papua New Guinea provided further evidence that *P. vivax* infection was associated with clinical protection against *P. falciparum* disease (11). In all of these studies, *P. falciparum* never protected against *P. vivax* infection.

The hypothesis that one species could suppress the pathogenicity of another (7) could also account for other unusual epidemiological observations from co-endemic areas. For example, distinct seasonal patterns characterized the incidence of *P. falciparum* and *P. vivax* in Vanuatu (13) and between *P. falciparum* and *P. malariae* in Nigeria (14), where each species was dominant at different times of the year and appeared to alter the infection dynamics of the other. Inter-species suppression of infection may also explain the recurrence of latent *P. vivax* or *P. malariae* following treatment of *P. falciparum* infections (15), and the low frequency of mixed infections in populations where multiple species co-exist (16, 17). In fact, this led to the suggestion by Cohen (16) that, “*If heterologous immunity can indeed greatly reduce the prevalence of mixed infections, as is claimed, then a malaria vaccine need not be specific to each of the species, strains, or antigenic variants of Plasmodium in order to be effective.*”

In none of these studies was there evidence that prior infection with one species reduced the risk of subsequent infection with another species. This is consistent with the lack of sterile immunity to any species of malaria, even against different strains within the same species. Rather, the evidence from these population-based studies suggests that the interactions among species may occasionally be protective and reduce the clinical course of disease. Even this cautious interpretation is subject to challenge by the many confounding factors that plague these types of epidemiological studies. It is very difficult to follow precisely the course of infection in individuals, even in longitudinal studies. This limitation is particularly apparent in light of the high frequency of submicroscopic infections revealed in more recent studies using molecular diagnostics (18). We cannot exclude persistent, submicroscopic infections of one species that could impact interpretation of these data. Alternative explanations to cross-species immunity have also been raised, including non-specific antiparasitic effects (19), ecological competition between parasites for the same mosquito host, and density-dependent mechanisms such as competition for red blood cells and nutrients within the human host (7, 16, 20). Given these limitations, we turn to studies with controlled infections in humans and laboratory animals to assess the validity of cross-species immunity.

## Experimental Human Infections

One of the earliest studies to deliberately infect human volunteers with *P. vivax* and *P. falciparum* was from the 1930s (21). Eight volunteers were infected with either *P. vivax* or *P. falciparum* from the bite of an infected mosquito then infected with the heterologous parasite either during the incubation period, the clinical phase, or following a recent infection with the first parasite. In the majority of these cases, the second infection was established, with no evidence of sterile immunity in these volunteers. Yet there was no discussion of whether the severity of symptoms during the second infection was affected by the primary infection.

The subsequent era of experimental human infections from 1940 to 1963 centered on malaria therapy treatments of patients with neurosyphilis. Treatment often resulted in multiple sequential infections with homologous or heterologous species, especially if the first treatment did not meet the therapeutic goals or due to limited availability of mosquitoes and patients infected with a particular species as a source of parasites for treatment. This may confound the interpretation of the results when comparing sequential infections to mono-infections since the reason for treatment failure is not known. Furthermore, homologous protection in control subjects was not always assessed. Despite this inherent variability, the malaria therapy cases yielded a wealth of data on the outcomes of infection with different species.

One of the earliest comprehensive reviews of these cases evaluated the effects of a primary malaria infection with *P. falciparum, P. ovale, P. malariae*, or *P. vivax* on patients re-infected with either the same or a different species (22). Upon homologous re-infection, the severity of the subsequent infection was significantly reduced but heterologous re-infections gave variable results. The outcome depended on the combination and order of species for the primary and secondary infections. In fifteen patients with a *P. vivax* infection followed by a *P. falciparum* infection, no effect on the second infection was observed when peak asexual parasitemia, gametocytemia and fever episodes were compared to single infections in malaria-naïve individuals. Similarly, there was no effect of a *P. malariae* infection on a subsequent *P. falciparum* infection (*n* = 6). However, previous infection with *P. vivax* led to lower parasite densities and fewer fever episodes during a subsequent *P. ovale* infection in 15 patients, compared to naïve individuals. When the order of these infections was reversed and *P. ovale* was given first, there was no effect on fever or parasitemia during the following *P. vivax* infection; however, the *P. vivax* infections were self-limiting, and no drug treatment was required.

There was a similar effect when a *P. falciparum* infection followed a *P. ovale* infection (*n* = 11). In these cases, there was obvious modification of the severity of the *P. falciparum* infection resulting in a much lower proportion of patients requiring treatment and in those that did, a lower therapeutic dose was sufficient. In fact, no curative doses of drugs were needed if the *P. falciparum* infection was preceded by a *P. ovale* infection. When the order of these infections was reversed in eleven patients, there was no effect of prior *P. falciparum* infection on the subsequent *P. ovale* infections. Only a small number of patients received a *P. vivax* infection after a *P. malariae* infection, but the *P. vivax* infection in 2 of 3 patients resolved spontaneously.

A later review of different patient files from the same time period suggested some cross-reactivity of *P. falciparum* with *P. malariae*, but not with *P. vivax* or *P. ovale* (23). The frequency of *P. falciparum* hyperparasitemia (≥ 10,000/μL) and fever was not affected by prior infection with *P. vivax* or *P. ovale* but was reduced when the *P. falciparum* infection was preceded by a *P. malariae* infection. This latter observation was countered in another review, concluding there was no evidence that past or current *P. malariae* infection affected *P. falciparum* asexual parasitemia; yet interestingly, there was an effect on *P. falciparum* gametocytemia (24). The authors proposed that the balance between asexual parasitemia and gametocytemia could be altered by the presence of the other species.

Collectively, the data from select human experimental studies bolster the evidence for cross-species interactions observed in the field studies, although this is clearly not a consistent occurrence. These studies further highlight the non-reciprocal nature of parasite interactions that appear to be predicated on the temporal sequence of infection and support a mechanism of partial heterologous immunity that can limit disease severity from the secondary infection.

## Infections in Experimental Animals

Animal models of malaria offer an analogous approach to investigate cross-species immunity in a controlled environment. Early studies that investigated interactions between *P. gallinaceum* and *P. lophurae* in chickens corroborated the findings of the various human studies that heterologous immunity can be non-reciprocal (25). Chickens infected with either *P. gallinaceum* sporozoites or blood stage parasites followed by infection with homologous parasites [as sporozoites or infected red blood cells (iRBCs)], or iRBCs of the heterologous species *P. lophurae*, exhibited marked reductions in both homologous and heterologous parasitemia. But when the order of the inoculations was reversed, weak heterologous immunity was observed and only in chickens that were hyperimmune to *P. lophurae* (following 4 or 5 infections).

There is ample evidence from mouse models that vaccination with attenuated sporozoites can elicit cross-species protection [reviewed in (26, 27)]. Mice immunized with X-irradiated *P. berghei* sporozoites were completely protected from heterologous challenge with *P. vinckei* sporozoites and immunization with irradiated *P. chabaudi* sporozoites induced sterile protection against infection with *P. berghei* (28). Furthermore, both irradiated and genetically attenuated *P. berghei* sporozoites inhibited intrahepatic development of *P. yoelii* sporozoites based on copies of parasite 18S ribosomal RNA quantified by qRT-PCR (29). Even immunization with *P. falciparum* sporozoites protected 60% of mice from a *P. berghei* infection (but not a *P. yoelii* infection) and passive transfer of IgG from these *P. falciparum* vaccinated mice protected naïve mice from a *P. berghei* sporozoite challenge (30). Similarly, chemically attenuated *P. berghei* sporozoites protected mice from challenge with *P. yoelii* sporozoites (31). In this case, cross-species protection was short-lived and did not last beyond 10 days post-immunization (31).

Likewise, blood stage murine parasites are capable of inducing cross-species immunity, which was reviewed extensively by Richie (7, 8). For instance, protection - measured as reduction in mortality - was observed when *P. berghei*-vaccinated mice were challenged with *P. yoelii* and when *P. vinckei*-vaccinated mice were challenged with *P. chabaudi* (32). Similar to Taliaferro and Taliaferro’s observations (25) with *P. gallinaceum* and *P. lophurae* in chickens, heterologous immunity in mice was non-reciprocal (32). Prior infection with *P. berghei* or vaccination with formalin-fixed blood-stage parasites reduced mortality in mice from *P. yoelii* infection, but no protection was observed when the species order was reversed. Similarly, mice vaccinated with *P. vinckei* were protected from *P. chabaudi* but not the inverse. One exception was the reciprocal cross-species protection between the blood stages of *P. berghei* and *P. vinckei.* After vaccination or infection with either parasite, 40–50% of mice survived a lethal heterologous challenge with the other species. From these studies and others, the genetic background of the mouse is likely to impact cross-protection. *P. chabaudi* immunization did not protect against *P. yoelii* challenge in outbred CD-1 mice (32), whereas partial protection was observed in BALB/c mice, and complete protection in C57BL/6 and CBA mice (33). More recently, this was observed using a different vaccination scheme termed ‘controlled infection immunization’ where mice were immunized with one species while under doxycycline chemoprophylaxis then challenged with the heterologous species (34). C57BL/6 mice immunized with *P. chabaudi* or *P. yoelii* promoted survival following heterologous challenge with the reciprocal parasite (34). While in BALB/c mice, protection was non-reciprocal; only *P. chabaudi* immunization could protect against *P. yoelii*, mirroring the findings from the older study.

Immunity in the studies described above was defined as protection from mortality, but as McColm and Dalton discuss (32), there is evidence of significant modulation of infection between species. This clinical suppression of disease was apparent as reduced parasitemia over the course of infection and delayed mortality relative to controls. Similarly, cerebral malaria was prevented in mice with a *P. berghei* ANKA infection if they had a co-infection with *P. yoelii* (but not with *P. vinckei* or *P. berghei* NK25) (35). It is important to note that in many of these studies the effects of non-specific anti-disease factors (such as cytokines or hormones) on secondary infections are impossible to separate from specific immune responses. Non-specific immune factors in sera from mice with a malaria infection inhibited *in vitro* growth of *P. falciparum* independent of antibody levels (36). Another factor may be hepcidin, which is upregulated in response to a blood stage malaria infection and inhibits a concurrent liver stage infection irrespective of the strain or *Plasmodium* species (37).

## Cross-Reactive Antibodies

The evidence supporting cross-species protection from human and animal studies validates efforts to explore heterologous vaccine strategies but also begs an understanding of the underlying immune mechanisms. Rather unexpectedly, insight into the immunological basis of cross-reactivity first emerged from attempts to develop species-specific diagnostic tests. In testing the specificity of a complement fixation assay for malaria diagnosis, Kingsbury detected cross-reactivity between *P. vivax* and *P. falciparum* antigens (38). Sera from 6 of 12 individuals with acute *P. vivax* infection reacted to *P. falciparum* antigens in a precipitin test, and likewise, 5 of 16 sera from patients infected with *P. falciparum* reacted to *P. vivax* antigens. However, a later paper by Mayer and Heidelberger (39) suggested that the specificity of the test was compromised by reactivity of sera with human stromata in the antigen preparations. In a different precipitin test developed by Taliaferro et al. (40, 41), sera from patients in Honduras infected with *P. vivax* reacted with antigens prepared from a *P. falciparum*-infected placenta. Surprisingly, heterologous reactions were as strong as the homologous ones. These findings were replicated in two separate studies in Honduras with over 500 sera but not in a later study in Puerto Rico with antigens prepared in the same manner (42). The results from the Puerto Rico study were deemed inconclusive and the inconsistency attributed to the generally poor performance of the precipitin test at that time. Serological cross-reactivity was later observed against *P. falciparum* and *P. vivax* crude antigens prepared from short-term culture of parasites isolated from infected patients, but the homologous reactions were more intense than the heterologous reactions (43).

With the advent of techniques to fluorescently label antibodies, their recognition of antigens from distinct malaria species could be directly observed under the microscope with the immunofluorescence assay (IFA). In one of the first records of this method applied to the study of human malaria, fluorescently labeled immunoglobulin from a patient with a long-standing *P. vivax* infection recognized RBCs infected with the simian malaria species *P. cynomolgi* (although not *P. berghei*) (44). This finding was replicated in another study where sera from *P. vivax*-infected patients (*n* = 4) recognized thin blood smears made from monkeys infected with *P. cynomolgi* (45). Homologous parasites were recognized much more strongly than heterologous parasites, but cross-reactivity in this case was reciprocal: serum from 5 volunteers infected with *P. cynomolgi* recognized two strains of *P. vivax* (Chesson and Venezuelan strains) by IFA using thin blood smears made from infected patients (45). Similarly, antibodies from a laboratory worker following an accidental *P. cynomolgi* infection recognized thin smears of *P. vivax* iRBCs as strongly as those infected with *P. cynomolgi* (44).

Immunological cross-reactivity between *P. vivax* and *P. falciparum* was also demonstrated with sera from naturally infected individuals (46). Sera from 9 out of 29 individuals with a *P. vivax* infection recognized *P. falciparum* iRBCs, while sera from 11 out of 21 individuals with a *P. falciparum* infection recognized *P. vivax* iRBCs. In this same study, cross-reactivity was also observed in individuals deliberately infected with *P. vivax* or *P. falciparum*. Based on the antibody titers against homologous versus heterologous iRBCs, *P. vivax* sera were more cross-reactive against *P. falciparum* iRBCs than the converse.

In Guatemala, sera from individuals naturally exposed to *P. vivax* strongly cross-reacted with asexual *P. falciparum* antigens by ELISA (20/43 positive), IFA (35/36 positive) and by immunoprecipitation assays (32/32 positive) (47). In order to rule out past *P. falciparum* infection as the source of these antibodies (despite > 99% prevalence of *P. vivax*), the sera were also tested against the *P. falciparum* CSP and heat shock protein (HSP) 70 kD-like-molecule repeat peptides by ELISA. Only 2 out of 36 sera samples recognized the PfCSP repeat peptide and 1 out of 33 sera samples recognized the *P. falciparum* HSP70 kD-like-molecule repeat peptide (48), suggesting the antibodies were truly cross-reactive. In this study, serological recognition of a HSP70 peptide was used to rule out antibodies specific to *P. falciparum* infection, but this family of proteins contains other epitopes that are shared across *Plasmodium* species (49). Given their ubiquitous nature, it is possible that these and other conserved housekeeping antigens underpinned some of the cross-reactivity discussed previously. While these may be viable targets of cross-reactive antibodies, their validity as vaccine candidates would depend on whether they elicit functional antibodies.

IFA was also useful to validate the interactions between the different species of rodent malaria and to develop a model of antigenic similarity among these parasites (50). Hyperimmune sera generated by infecting mice three times with either *P. berghei, P. yoelii, P. chabaudi*, or *P. vinckei* revealed that the four species were serologically indistinguishable by IFA. Sera from rabbits immunized with soluble antigens from these parasites gave similar results. These findings form the basis of a proposed model of antigenic conservation between the four murine malaria species whereby certain antigens are shared among all four species, some antigens are shared only between the most similar pairs of parasites and then others are specific to each species (50).

## Cross-Reactive T-Cells

Cellular immunity is also likely to play a role in cross-species immunity and may underpin the protective clinical effects (reduced symptoms and disease severity) - yet there is scant data on the potential contribution of T-cells to this immune mechanism. In rodent models, antibody-independent mechanisms clearly influenced susceptibility to heterologous challenge (51). For example, B-cell deficient mice chronically infected with *P. yoelii* were resistant to lethal challenge with *P. chabaudi* (51). Cross-reactive T-cell responses are also vital to the heterologous immunity observed in murine malaria models of attenuated sporozoite vaccination. Immunization with radiation-attenuated *P. berghei* sporozoites protected 79% of mice challenged with *P. yoelii* sporozoites and immunization with *P. yoelii* sporozoites protected 63% of mice challenged with *P. berghei* sporozoites (52). Heterologous protection was dependent on CD8^+^ T-cells whereas antibodies from immunized mice only recognized homologous, but not heterologous, sporozoites. In another study, 100% of mice immunized with genetically attenuated *P. yoelii* sporozoites were protected against *P. berghei* sporozoite challenge (53). The authors suggested that late-liver stage arresting sporozoites elicited a broadly protective CD8^+^ T-cell response.

There are few reports of species-transcending T-cells in humans. The most definitive study showed that T-cells isolated from volunteers immunized with attenuated blood stage *P. falciparum* parasites proliferated *in vitro* in response to *P. knowlesi* iRBCs (54). Whether these T-cells have functional activity to protect against heterologous challenge is not known.

## Potential Vaccine Targets

The data presented in this review build the case for heterologous immunity elicited by natural or deliberate infection in animals and humans. The mechanism of immunity likely involves both humoral and cellular immunity, but the antigenic determinants are unknown. To translate the observations spanning the last century into viable heterologous vaccine candidates, the conserved targets of immunity must be identified. Given that partial protection is observed in nature and appears to be a relatively rare event, we expect that multiple vaccine candidates will be needed to target different stages of the parasite’s lifecycle. Cross-species vaccines that aim to prevent infection should target sporozoite antigens to inhibit hepatocyte invasion and development, while vaccines that target blood stage antigens could prevent severe disease. Gametocyte antigens are also attractive targets to prevent the onwards transmission of malaria. Below, we review the discovery and current knowledge of potential cross-species antigens from different parasite stages. We summarized the findings from studies using human malaria parasite antigens in Figure 1.

### Pre-erythrocytic Targets

The focus of immunity to sporozoite/liver stage infection is largely on CSP. Cross-reactive immune responses between CSP from *P. vivax* and *P. falciparum* have been reported in both naturally exposed populations (55) and in controlled human malaria infections (CHMI) (56). In populations from a region in Brazil endemic for both species, peripheral blood mononuclear cells (PBMCs) were highly responsive to stimulation with either PfCSP or PvCSP (55). Responses to both species were especially frequent in individuals recovering from a recent *P. vivax* infection; PBMCs from 35 to 54% of these individuals proliferated in response to PfCSP (55). These findings suggested that PvCSP and PfCSP might share cross-reactive T-cell epitopes, while there was no evidence of heterologous antibody responses. In contrast, deliberate infection of naïve volunteers with either *P. falciparum* or *P. vivax* by mosquito bite gave rise to heterologous antibody responses to CSP from each species (56). In both groups, 61% of volunteers had antibodies that cross-reacted with the heterologous CSP antigen. These heterologous responses were largely mediated by IgM and not IgG.

Only one study supports a role for CSP in cross-species protection, a key criterion for pursuing CSP as a heterologous vaccine target. In mice, a CD8^+^ T-cell clone generated through vaccination with irradiated *P. yoelii* sporozoites recognized a peptide from PyCSP and the homologous peptide from *P. berghei* PbCSP (57). Adoptive transfer of this clone to naïve mice protected against homologous (*P. yoelii*) and heterologous (*P. berghei*) sporozoite challenge. The specificity of the T-cell epitope appears to be critical for cross-species immunity. In another study, mice were immunized with attenuated *P. berghei* sporozoites and CD8^+^ T-cells that recognized a different peptide from PbCSP were selected and transferred into naïve mice (58). These CD8^+^ T-cells only recognized the peptide from PbCSP and not a related peptide from PyCSP that differed in sequence at three amino acid positions. Consistently, the recipient mice were only protected against a homologous challenge with *P. berghei* sporozoites, and not against heterologous challenge with *P. yoelii* sporozoites. Similar species-specific T-cell responses were observed in response to vaccination with *P. yoelii* antigens (59, 60). Mice were immunized with a T-cell epitope from PyCSP and lymph node cells isolated from these mice specifically inhibited development of *P. yoelii* liver stage schizonts *in vitro*, but not *P. berghei* schizonts (59). However, immunization with this epitope did not significantly protect mice against a homologous sporozoite challenge. In a follow-up report to this study, Rénia et al. (61) demonstrated homologous protection by passively transferring peptide-specific T-cell clones to naïve mice. They failed to observe inhibitory cross-reactivity of these T-cells against *P. berghei* sporozoites *in vitro* and therefore did not test for cross-reactivity *in vivo*.

Consistent with the lack of protection observed in some of the rodent studies, immunization of mice with *P. falciparum* PfCSP conferred no protection against heterologous challenge with *P. berghei* sporozoites (30). Similarly, immunization with B-cell epitopes from the *P. falciparum* and *P. yoelii* CSPs inhibited homologous sporozoite invasion *in vitro* but had no effect against heterologous sporozoites (62). It is perhaps not surprising that cross-species immunity is not mediated through PfCSP. The RTS,S vaccine based on PfCSP is poorly boosted by natural infection and fails to elicit robust strain-transcending immunity, with no prospects for species-transcending immunity since the CSP repeats in each species are very different (63). However, it is possible that PvCSP could prove a better candidate. Sera from mice immunized with a PvCSP vaccine candidate recognized both *P. falciparum* and *P. berghei* sporozoites by IFA (63). Immunized mice were also protected from a *P. berghei* infection initiated by the bite of an infected mosquito. As described earlier, cross-species immunity is mostly non-reciprocal and in many of the studies reviewed here, *P. vivax* confers broader cross-reactivity compared with *P. falciparum*.

Other liver stage antigens are potential targets for a cross-reactive vaccine. Sera from mice immunized with the *P. falciparum* cell-traversal protein for ookinetes and sporozoites (PfCelTOS) recognized *P. berghei* sporozoites by IFA and protected 60% of BALB/c mice from infection with *P. berghei* sporozoites (64). However, cross-species protection was not observed when PfCelTOS was expressed from a viral vector (65). The *uis3* gene represents another potential liver stage target and is conserved across human, primate and rodent *Plasmodium* species. It is actively transcribed in sporozoites but translationally repressed until the parasite infects hepatocytes (66). Mice immunized with PfUIS3 and challenged with *P. berghei* sporozoites exhibited a significant delay in the time to patent parasitemia (65). It should be noted that PbUIS3 was not specifically shown to mediate this cross-species protection and a search for predicted cross-reactive linear epitopes did not reveal peptides with high conservation between the two orthologs. PfLSA3, another sporozoite and liver stage antigen with unknown function, elicits cross-reactive antibody and cellular immune responses against rodent malaria (67, 68). PfLSA3-specific antibodies purified from hyperimmune human sera or from an immunized chimpanzee recognized *P. yoelii* sporozoites by IFA and western blot, blocked invasion of murine hepatocytes by *P. yoelii* sporozoites and protected mice from *P. yoelii* challenge in a pilot experiment (*n* = 4). The epitopes shared between the *P. falciparum* and *P. yoelii* proteins enable reciprocal immune recognition as antibodies from mice infected with *P. yoelii* recognized peptides from PfLSA3 yet given the absence of a PfLSA3 ortholog in *P. yoelii*, these antibodies may be targeting another related antigen or the cross-reactivity is not specific. Furthermore, this cross-reactivity is restricted to *P. yoelii*, as sera from mice infected with *P. berghei* did not cross-react with PfLSA3 and likewise, human PfLSA3 antibodies failed to recognize *P. berghei* sporozoites or block invasion of hepatocytes.

### Erythrocytic Targets

The merozoite surface protein (MSP) family includes several blood stage vaccine candidates whose homology across different *Plasmodium* species may be exploited for a cross-species vaccine. For example, IgG responses from a subset of individuals in Indonesia were cross-reactive to both merozoite surface proteins PfMSP5 and PvMSP5 (69). Sera from 82 individuals with a *P. falciparum* infection, 85 individuals with a *P. vivax* infection, 85 individuals with mixed infections and 87 exposed, but asymptomatic individuals, were tested by ELISA. Of these, 107 dual-positive responders were identified that recognized both PfMSP5 and PvMSP5. Using competition ELISAs, 7 samples were identified as truly cross-reactive; in other words, recognition of MSP5 from either species could be blocked by pre-incubation with the MSP5 from the other species. Although the overall frequency of cross-reactivity to these two proteins was low (7%), these findings suggest that a vaccine that targets the cross-reactive epitopes may protect against more than one *Plasmodium* species.

MSP-1 is another viable blood stage candidate. In the CHMI study described earlier (56), 50% of volunteers infected with *P. vivax* had antibodies to PfMSP-1 while 67% of those infected with *P. falciparum* recognized PvMSP-1 on day 28 after infection. Far lower frequencies of cross-reactivity were observed in other studies. Using a multiplex bead assay, the species specificity of IgG responses to the MSP1_19_ region from *P. falciparum, P. ovale, P. vivax*, and *P. malariae* was evaluated in sera from experimentally infected chimpanzees, infected individuals living in low transmission settings in Haiti and Cambodia (*n* = 12), and sera eluted from blood spots collected from individuals living in a high transmission setting in Mozambique (*n* = 20) (70). All of the antibody responses from the chimpanzees were species-specific and recognition was completely blocked by competition with the homologous protein. Only one out of 12 samples from people living in the low transmission setting and 8 of 20 samples from people living in high transmission settings showed partial, and highly heterogeneous cross-reactivity to select other species. Cross-reactivity was mostly non-reciprocal and there were very few sera that cross-reacted with all four species of *Plasmodium*.

Another pair of orthologs that share B-cell epitopes is PfCLAG9 and PvCLAG7 (71). These proteins localize to the rhoptries and play a role in erythrocyte invasion. In this study, cross-reactive antibodies were observed in naturally exposed populations in the Brazilian Amazon and these findings were modeled in mice. Antibodies from mice immunized with PfCLAG9 peptides exhibited very similar surface staining of RBCs infected with either *P. vivax* or *P. falciparum* by IFA. The functional activity of these antibodies against the heterologous parasite was not reported.

Antigens that elicit cross-reactive antibodies against orthologous proteins from non-human and human *Plasmodium* species would be attractive vaccine candidates since their broad conservation across the evolutionary spectrum of these parasites implicates these proteins in parasite survival. Recombinant *P. falciparum* HGPXRT stimulated mouse CD4^+^ T-cells primed with the *P. yoelii* ortholog, implying these proteins share T-cell epitopes (72). *In vivo*, mice immunized with *P. falciparum* HGPXRT controlled parasitemia and induced partial protection against a *P. yoelii* challenge. Another promising group of proteins are the merozoite-released soluble proteins (MRSPs) (73). Mice immunized with the *P. falciparum* MRSPs were protected against a blood stage *P. yoelii* infection and IgG purified from mice infected with *P. yoelii* inhibited *P. falciparum* growth and invasion *in vitro.* Furthermore, *P. falciparum* MRSPs bound to mouse erythrocytes and *P. yoelii* MRSPs bound to human erythrocytes, suggesting conservation of functionally related proteins across the two species.

Apical membrane antigen 1 (AMA1) is another viable cross-species vaccine candidate; it is an invasion protein present in many *Plasmodium* species and there is strong evidence of structural and functional conservation between orthologs (74). In fact, PvAMA1 replaced PfAMA1 in transgenic parasites without compromising parasite growth (74). Polyclonal rabbit antibodies raised against either antigen recognized the heterologous protein by western blot and stained the parasites by IFA. Further support for immunological cross-reactivity among these proteins stems from epitope mapping studies with a monoclonal antibody (mAb) raised against PvAMA1 that recognized AMA1 from *P. knowlesi, P. falciparum, P. cynomolgi* and *P. berghei* by IFA (75). Co-crystallization of the mAb with either PvAMA1 or PfAMA1 revealed striking structural similarity between the epitopes in both proteins and most of the contact residues were conserved. Interestingly, this conservation extends to the AMA1 orthologs from the other *Plasmodium* species recognized by the same mAb. It is important to note that in both of these studies on AMA1, there was evidence of antibody cross-reactivity, but these antibodies were not functional. The rabbit sera against PfAMA1 did not block RBC invasion by the *P. falciparum* transgenic strain that expressed PvAMA1 (74). Likewise, the PvAMA1 mAb recognized *P. cynomolgi* but did not block invasion by this species *in vitro* (75). This may be due to lower avidity of the PvAMA1 mAb against the heterologous antigens, as shown by surface plasmon resonance (SPR) with PfAMA1. These data provide a starting point to design a cross-species vaccine against AMA1 but emphasize the importance of defining epitopes that will yield inhibitory antibodies against the heterologous species.

### Transmission-Blocking Targets

The goal of a transmission-blocking vaccine is to disrupt the life cycle of the parasite by interrupting transmission to the mosquito. A vaccine with the potential to achieve this across multiple species would be a pivotal public health tool to support malaria elimination. To the best of our knowledge, only the gametocyte antigen P48/45 has emerged as a candidate for cross-species recognition. Sera from school-aged children living in a *P. falciparum* endemic area of Zimbabwe were highly cross-reactive to the *P. vivax* homolog Pvs48/45 (76). Thirty-six of 49 (73%) samples positive for Pfs48/45 by ELISA also recognized Pvs48/45. These results were confirmed by western blot on 23 randomly selected samples. Officially, there was no *P. vivax* transmission in the area at the time of sample collection (2015), but the authors detected one low-level *P. vivax* infection out of 27 randomly selected blood samples that were tested by nested PCR, suggesting a potential caveat to these findings. Nonetheless, similar results were observed in mouse models (77). Sera from mice immunized with recombinant Pfs48/45 or Pvs48/45 recognized the heterologous proteins by ELISA and IFA. Importantly, the antibody responses against heterologous antigens were cross-boosted; for example, mice immunized once with Pfs48/45 then boosted with Pvs48/45 rapidly acquired anti-Pfs48/45 antibodies that were not present following the primary immunization. This strongly implicates that specific B-cell epitopes are conserved across the orthologous proteins. A vaccine based on these epitopes that could be boosted by natural infection with either species would be a powerful intervention against malaria.

### Cross-Species Immunity to Heterologous Proteins

**FIGURE 2:**
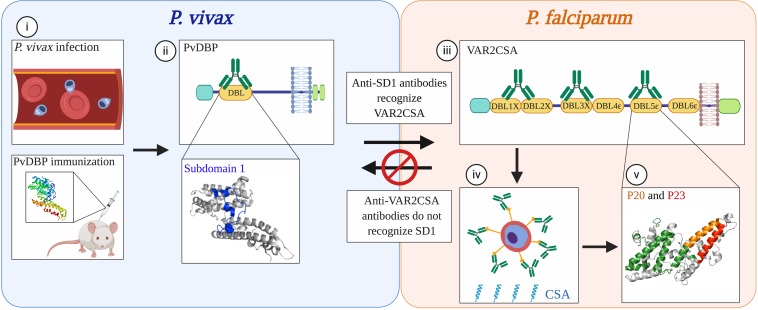
Non-reciprocal, cross-species immunity mediated by conserved domains in functionally distinct proteins from *P. vivax* and *P. falciparum*. (i) Antibodies to the *P. vivax* merozoite protein PvDBP that arise from natural infection in humans or by vaccination with the recombinant protein in mice recognize epitopes within the DBL domain of PvDBP (ii). A subset of antibodies (green) that recognize subdomain 1 (SD1; blue) also cross-react with the DBL domains of *P. falciparum* VAR2CSA (iii), a protein that mediates sequestration of parasites in the placenta. Although PvDBP is not thought to play a role in *P. vivax* pregnancy-associated malaria, antibodies against the SD1 region of PvDBP can block *P. falciparum* parasites from binding *in vitro* to CSA, the placental ligand (iv). The recognition sites of these cross-reactive antibodies in one of the DBL domains of VAR2CSA, DBL5ε, map to two non-overlapping peptides, P20 (orange) and P23 (red) (v). These epitopes are spatially distinct from the immunodominant epitopes recognized by sera from multigravid women from Tanzania (green). Concordantly, these epitopes are cryptic; P20 and P23 are not recognized by sera from multigravid women from Uganda, nor by sera from rabbits immunized with VAR2CSA. As observed in other studies of human and mouse malaria, the immune recognition of these proteins is non-reciprocal as antibodies elicited through natural exposure to VAR2CSA in pregnant women or through immunization of animals with recombinant VAR2CSA did not recognize SD1. The cross-reactivity of antibodies to PvDBP and VAR2CSA exemplifies a mechanism of immune recognition to functionally distinct proteins in different *Plasmodium* species that is mediated by structurally conserved domains. Modified from (79). Created with Biorender.com.

All the studies considered above investigated cross-reactivity between orthologous proteins yet there is evidence (although sparse) of cross-reactivity between functionally unrelated antigens from different species. We demonstrated immunological cross-reactivity between heterologous proteins in *P. vivax* and *P. falciparum* (78, 79) (Figure 2). Based on the unexpected finding that Colombian men and children had antibodies to the pregnancy-specific *P. falciparum* antigen VAR2CSA, we discovered that prior exposure to *P. vivax* Duffy Binding Protein (PvDBP) can give rise to antibodies that cross-react with VAR2CSA (78). We further mapped an epitope in the Duffy Binding Like (DBL) domain of PvDBP that mediates this cross-reactivity (79). Human antibodies affinity-purified against this epitope can block adhesion *in vitro* of VAR2CSA-expressing iRBCs to the placental receptor chondroitin sulfate A (CSA). The surprising aspect to this immune pathway is that PvDBP and VAR2CSA are not functionally related. Rather, they have a common homologous ancestor which gave rise to conserved structural features shared among a large family of erythrocyte binding proteins (80). These structural domains are ubiquitous in human and rodent *Plasmodium* species. However, the immunogenicity of cross-reactive epitopes within these proteins is likely variable across species. In *P. vivax*, the cross-reactive epitope is subdominant, arising only in a subset of exposed individuals (79, 81). In turn, these antibodies target epitopes in VAR2CSA that are cryptic, and reciprocal immunity is not elicited by exposure to VAR2CSA in pregnancy or by vaccination (79). Cross-reactivity is therefore a rare event – but once identified, can be exploited for vaccine design.

It is even conceivable that a vaccine could be designed to confer protection across species and across parasite stages. A 60 amino acid peptide based on a cryptic epitope discovered in PfCSP elicited antibodies in mice that recognized asexual blood stages of both *P. falciparum* and *P. yoelii* by IFA and blocked *P. falciparum* merozoite invasion by 70% *in vitro* (82). Strikingly, more than 60% of mice immunized with the PfCSP peptide survived a lethal blood stage infection with *P. yoelii* (although only 2 control animals were included). The authors reported that the PfCSP anti-peptide sera recognized a 60–65 kDa parasite protein in *P. falciparum* blood stage lysates. This protein may be related to TRAP, a protein expressed during liver and blood stages that shares amino acid similarity to the PfCSP peptide sequence. Importantly, this study demonstrated that a peptide vaccine based on a cryptic epitope can focus the immune response on conserved regions of the protein, with the potential to target related antigens in other stages of the parasite life cycle.

## Discussion

### The Rationale for a Cross-Species Vaccine

These studies revealed that individual antigens can elicit cross-reactive immune responses. However, the lack of sterilizing immunity to malaria during a lifetime of natural infection implies that a multivalent vaccine would be needed to provide cross-species protection. It is conceivable that the whole parasite vaccine approach could replicate the partial cross-species immunity observed in the studies discussed above. Parasites attenuated by irradiation, chemical treatment or genetic modification expose the immune system to a broad spectrum of antigens for that given stage, including antigens that are highly conserved across species (e.g. housekeeping antigens). If these attenuated parasites can persist in vaccinated individuals and remain metabolically active (83), this creates an opportunity for sustained antigenic stimulation of either B- or T-cells with the potential for more robust protection from future infection. In an older review of CHMI studies, only one volunteer was immunized with radiation-attenuated *P. falciparum* sporozoites and challenged with *P. vivax*. This person was not protected from vivax infection (84). Further CHMI studies are needed to test for cross-species immunity.

An alternative vaccine approach is to define the conserved epitopes in related antigens from different species and focus the immune response on these epitopes. This hinges on the hypothesis that despite the extreme antigenic diversity in *Plasmodium*, there exist evolutionarily conserved epitopes that can elicit protective antibodies or stimulate cross-reactive T-cells. Richie argued that selection pressure would favor antigenic diversity in species that infect the same host to avoid cross-species immunity that could eliminate both species (7). The continued scourge of malaria globally supports this tenet.

It is clear from the analysis of *Plasmodium* genomes that the immunodominant antigens in all species are highly polymorphic. Yet if we consider the functions of these diverse antigens, they are largely restricted to the pathogenesis of a particular species. For instance, the PfEMP1 virulence factors mediate sequestration of iRBCs to different tissues as a mechanism of immune evasion. Sequestration may have evolved to enhance the virulence of *P. falciparum* over other species that co-circulate in a given population and compete for the same host. But since the PfEMP1 family is unique to *P. falciparum* (and *P. reichenowi* in primates), the diversity among the members of the PfEMP1 family hinders the acquisition of strain-transcending immunity only within this species. This is also exemplified by the highly polymorphic proteins involved in erythrocyte invasion. *Plasmodium* species exhibit host cell tropism for different types of RBCs and evolved parasite ligands that bind to host receptors on those specific cells. In *P. vivax*, the PvDBP ligand interacts with the Duffy antigen receptor for chemokines (DARC) to invade reticulocytes; there is extensive diversity in the PvDBP domain that interacts with DARC. These polymorphisms are selected to evade immune responses that would block invasion of reticulocytes, yet they remain specific to *P. vivax* and do not impact other species that require different ligand-receptor interactions for invasion.

Despite the selection for variation in the immunodominant antigens within each species, many of these proteins evolved from ancestral homologs. The PfEMP1 and PvDBP proteins share a common protein architecture which includes DBL domains. While the functions of proteins with DBL domains diverged significantly within and across species, these domains may nevertheless have conserved epitopes that are essentially ‘evolutionary relics’. These epitopes are probably not highly immunogenic and would induce antibodies with lower avidity toward their heterologous counterparts. They may even be cryptic in some proteins, which could explain the non-reciprocal nature of cross-species immunity observed in so many of the human and animal studies (Figure 2). Based on the data reviewed here, we propose that these epitopes may be more exposed in less virulent parasites and cryptic in more virulent ones. This could provide a competitive advantage for the benign parasite and ensure survival of the host.

### Strategies and Challenges

**FIGURE 3:**
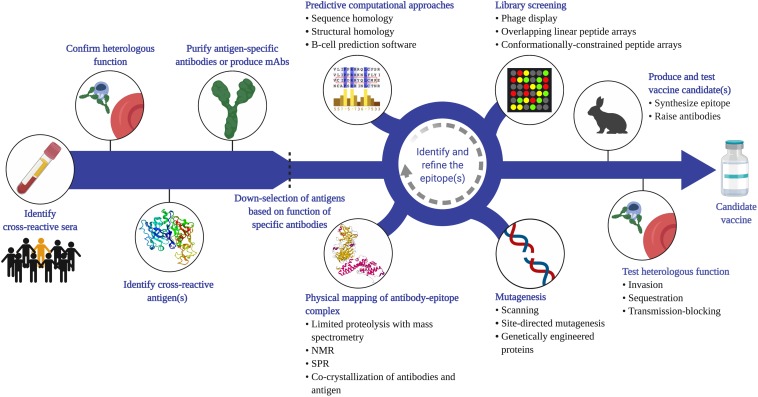
Proposed strategy to identify cross-species vaccine candidates based on cross-reactive B-cell epitopes. Given that cross-species immunity is a rare event in naturally exposed populations, a large number of samples from endemic populations will need to be screened (e.g. by IFA or flow cytometry) to identify sera that recognize heterologous parasites. Antibody function against the heterologous species should then be confirmed (e.g. invasion, cytoadherence, transmission-blocking assays), and the antigen that mediates this functional cross-reactivity identified. This can be achieved through a variety of methods, including depletion or competition experiments. Antigen-specific antibodies can be affinity-purified from sera, or monoclonal antibodies generated using PBMCs from naturally exposed individuals or from animals vaccinated with the antigen. Functional analysis of these antibodies can then be used to down-select candidate antigens before applying a variety of empirical approaches to map the cross-reactive epitope. Phage and peptide libraries can be screened with the cross-reactive antibodies. Mutagenesis techniques, such as site-directed mutagenesis or alanine scanning of recombinant proteins can map residues that are critical for antibody binding. Physical mapping, such as co-crystallization of the antigen-antibody complex, is another powerful approach to map the contact residues within the epitope. These experimental tools can be integrated with computational analysis of the antigens from each species. Once a putative cross-reactive epitope is identified, the next step is to generate a recombinant protein or synthetic peptide that recapitulates this epitope, raise epitope-specific antibodies in animals, and test for cross-reactivity and function *in vitro.* It is important to note that the process of identifying and refining the epitope is iterative and each approach can complement and inform the other to yield potent, functional cross-species antibodies. Created with Biorender.com, including crystal structures PDB accession numbers 1SME and 6R2S.

We propose that vaccination can refine and amplify a cross-species immune response to target heterologous antigens. There are a number of potential vaccine targets identified already that elicit cross-reactive antibodies (Figure 1) and certainly new targets to discover (Figure 3). The first step to identify new cross-reactive B-cell epitopes is to test sera for reactivity to heterologous parasites (e.g. by IFA). Cross-reactive sera should then be assessed for functional activity against the heterologous parasite. This could involve testing the sera in various *in vitro* assays to measure effects on invasion, sequestration, transmission-blocking activity, etc. Most of these assays measure antibody function, with only indirect assays to measure T-cell mediated responses (e.g. cytokine production) (85, 86). Even the more established antibody-based assays, such as the growth inhibition assay, vary in terms of validity and predictive value, and are largely antigen and strain-specific (87). Assays to measure adhesion-blocking activity also vary with the format; for example, the anti-adhesion activity of VAR2CSA antibodies varies significantly when compared using a static inhibition of binding assay, a flow-based assay, and a placental perfusion assay (88). Nevertheless, these assays can provide insight into the pathway blocked by those antibodies and generate hypotheses of which antigens are likely targets. Once the target protein is identified (through biochemical and/or immunological methods), antibodies specific to this antigen can be purified from the sera or generated as mAbs. These antibodies can be characterized in terms of their cross-reactivity (titers, avidity) and their functional activity against the heterologous parasite.

To translate these findings into vaccine candidates, the cross-reactive epitope can be mapped using a variety of approaches. B-cell epitopes may be linear but are more likely to be conformational if they represent structurally related epitopes. Conformational epitopes are certainly more challenging to map but advances in structural and computational biology provide valuable tools that support rational vaccine design [reviewed in (89)]. For example, cross-reactive human or mouse mAbs can be co-crystallized with the target protein to map the epitope empirically (75). These antibodies can also be used to screen peptide libraries (conformationally constrained or linear) or tested against mutant recombinant proteins to identify the epitope that mediates cross-reactivity. In parallel, computational approaches can be applied to protein databases to predict conserved epitopes. This technique was recently adopted to predict conserved linear and discontinuous epitopes in CSP and MSP-1 shared between the *P. falciparum* and *P. vivax* orthologs (56). Computational modeling can also guide the formulation of vaccines to enhance immunogenicity and to elicit broadly neutralizing antibodies. Computational simulations of affinity maturation applied to the antibody response to *P. falciparum* AMA-1 revealed that polyvalent vaccines promoted cross-reactive antibody responses to shared epitopes (across strains; AMA-1 from different species was not included) (90). In future, entire proteomes and epitope libraries spanning the evolutionary spectrum of Plasmodia can be probed using artificial intelligence and machine learning to discover targets of cross-species epitopes. It is important to note that the process of identifying and refining the epitope is iterative and each approach can complement and inform the other.

Once a cross-species epitope is mapped, the next step is to reproduce the epitope synthetically such that it can elicit functional antibodies or protective cellular responses against the heterologous epitopes in other species. This can be achieved with engineered recombinant proteins that expose the epitope preferentially (e.g. 91), linear epitopes conjugated to carrier peptides, and for conformational epitopes, this is feasible with the use of peptide scaffolds that restrict the conformation of peptides as immunogens (e.g. 92). An alternative delivery platform is the use of transgenic parasites from one species engineered to express antigens from a different species (93). The success of these approaches will depend on the fine specificity of the antibodies and their avidity for the heterologous epitopes. The avidity of cross-species antibodies observed in human and animal infections is generally low but different immunization strategies can be adopted to promote affinity-maturation, including the choice of adjuvant, delivery platform, dosing, and boosting schemes (2). As such, several rounds of identification, designing and testing may be required to produce potent, functional cross-species antibodies.

One outstanding question is whether these immune responses would be boosted by natural infection with heterologous species. This may depend on a number of variables including the intensity of parasite transmission, host genetics, and immune regulation. We hypothesize that if the epitope is truly cross-reactive, then memory B- or T-cells may be expanded by exposure to the heterologous epitope even if it is not immunogenic in that species. This phenomenon was recently reported for a cryptic epitope in group A streptococcus where immunization with the conserved peptide was boosted by natural infection with different bacterial strains (94). Similar vaccine strategies are being adopted against cryptic epitopes in Ebola antigens (95), and toward the development of a universal influenza vaccine (96–98).

## Conclusion

The slow progress in developing a malaria vaccine underscores the many challenges with a traditional vaccine approach. We need to consider alternative, yet complementary strategies. Thus, exploiting rare immune mechanisms like cross-species immunity are worthy of consideration and with our current tools, this is more amenable than ever before. We don’t expect this approach to yield a vaccine that provides sterile immunity to malaria; but if we could emulate the reduction in disease severity observed with heterologous infections in humans and in animal studies, this vaccine could reduce mortality in the most vulnerable populations and allow natural, strain-transcending immunity to develop.

## Author Contributions

CM and SY jointly wrote the manuscript.

## Conflict of Interest

The authors declare that the research was conducted in the absence of any commercial or financial relationships that could be construed as a potential conflict of interest.
